# Primary clear cell adenocarcinoma of the bladder with recurrence: a case report and literature review

**DOI:** 10.1186/1477-7819-10-33

**Published:** 2012-02-10

**Authors:** Ji Lu, Zhihui Xu, Fengming Jiang, Yan Wang, Yuchuan Hou, Chunxi Wang, Qihui Chen

**Affiliations:** 1 Department of Urology, The First Hospital of Jilin University, Changchun, China; 2 The Key Laboratory of Pathobiology, Ministry of Education, School of Basic Medical Sciences, Jilin University, Changchun, China

**Keywords:** Bladder, clear cell adenocarcinoma, recurrence

## Abstract

Clear cell carcinoma of the bladder is a rare tumor of the bladder. There are few reports available on this rare disease, and no cases with recurrence were reported. Here we present a case of 68-year-old woman with primary clear cell carcinoma of the bladder, who underwent repeat TUR-BT and tumor recurrence. We also reviewed the previous treatments and prognoses in previous case reports and evaluate the proper treatment for this disease. Once the diagnosis is determined, the radical surgery should be recommended. The recurrence is not prevented based on post-TUR intravesical therapy.

## Background

Bladder cancer is the most common malignancy of the urinary tract, and the 9^th ^most common cancer diagnosis worldwide, with more than 330,000 new cases each year and more than 130,000 deaths per year. Its generally estimated male:female incidence ratio is 3.8:1.0 [1]. At any point in time 2.7 million people have a history of urinary bladder cancer [1]. The histological and pathological type of bladder cancer is mainly urothelial carcinoma, also called transitional cell carcinoma, accounting for approximately 90%[2]. Other types including squamous cell carcinoma and adenocarcinoma, account for 3-7% and < 2% respectively [3]. Based on the definition of WHO, bladder adenocarcinoma is a malignant neoplasm derived from the urothelium showing histologically pure glandular phenotype [4]. Clear cell type is one of its histological growth patterns. Other patterns include enteric (colonic) type, adenocarcinoma not otherwise specified (NOS), signet ring cell, mucinous (colloid), hepatoid and mixed [4]. As an extremely rare variant of adenocarcinoma, clear cell type affects women more often than men. There are few reports available on this rare disease, and no cases with recurrence were reported. In the present report, a case of clear cell carcinoma of the bladder with repeat recurrence is presented. The purpose of our article is to add an additional case to the literature, to review the literature, and to formulate treatment recommendations.

## Case Presentation

**Figure 1:**
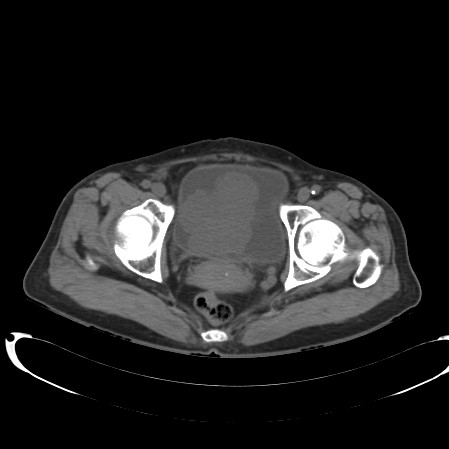
**CT showed a tumor at posterior wall of the bladder which has not invaded the perivesical spaces and uterus**.

A 68-year-old female was admitted to our department with 1-year history of dysuria and painful urination. The symptoms were partially relieved after antibiotic therapy, but had become worse in the previous 2 months, accompanied by urinary frequency and urgency. No positive physical signs were found. HGB in blood was 98 g/L. Routine urinalysis showed that RBC in urine was 210.80/ul and WBC in urine was 4277.40/ul. Other routine test results were normal. Ultrasonography demonstrated a 6.0 cm × 7.4 cm mass with low-level echo in the bladder, with an irregular shape and rich blood supply. On CT the inferior and posterior wall had become thickened and from which a 6.4 cm × 7.4 cm mass protruded into the bladder cavity and the urethra. CT also showed that the mass did not invade into the uterus and no pelvic lymphadenopathy was detected. (Figure 1). Cystoscopy demonstrated a mass measuring about 6 cm in maximum diameter with cauliflower-like surface located in the right lateral wall and extending to the internal urethral orifice. The right ureteric orifice was invisible. The patient was diagnosed with bladder cancer, urinary tract infection and anemia. The tumoral mass was resected by means of transurethral resection (TUR) and all visible masses were completely resected.

**Figure 2:**
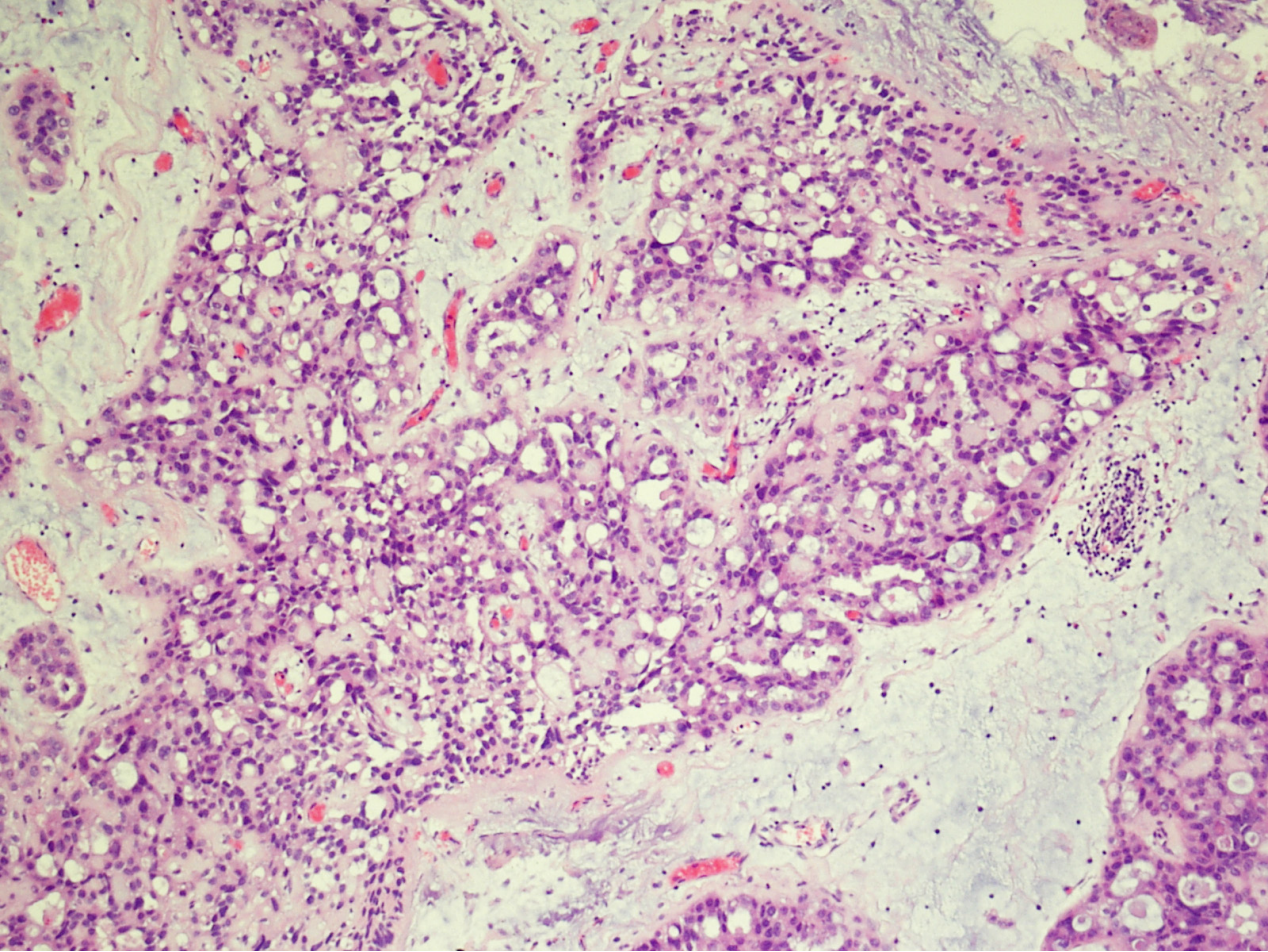
**Tumour cells were arranged in tubular gland, microcysts or fine papillary in most area, partly in diffuse sheets or solid nests**.

**Figure 3:**
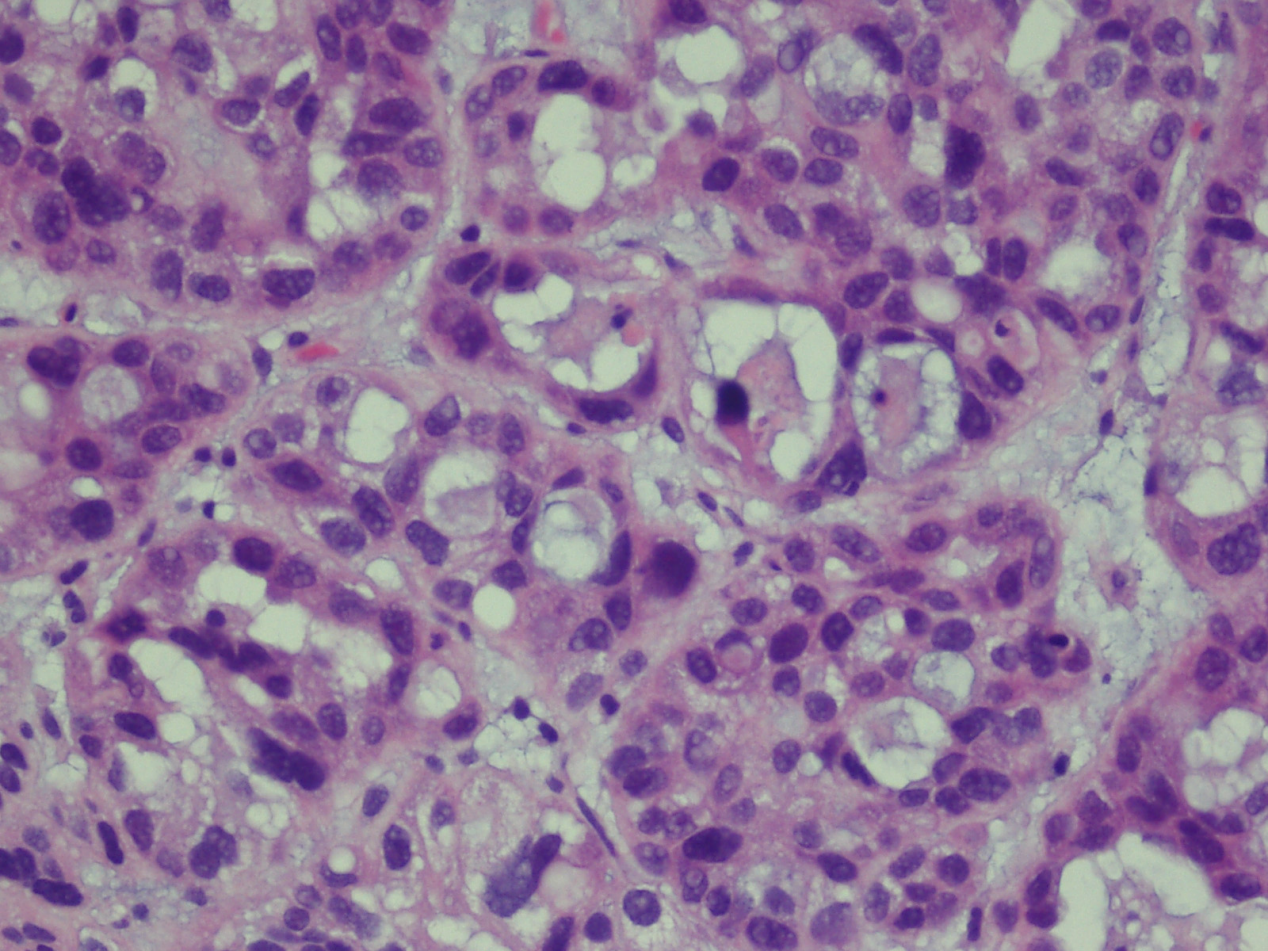
**Tubular glands and microcysts have different sizes and irregular shapes with eosinophilic and/or basophilic secretions**.

**Figure 4:**
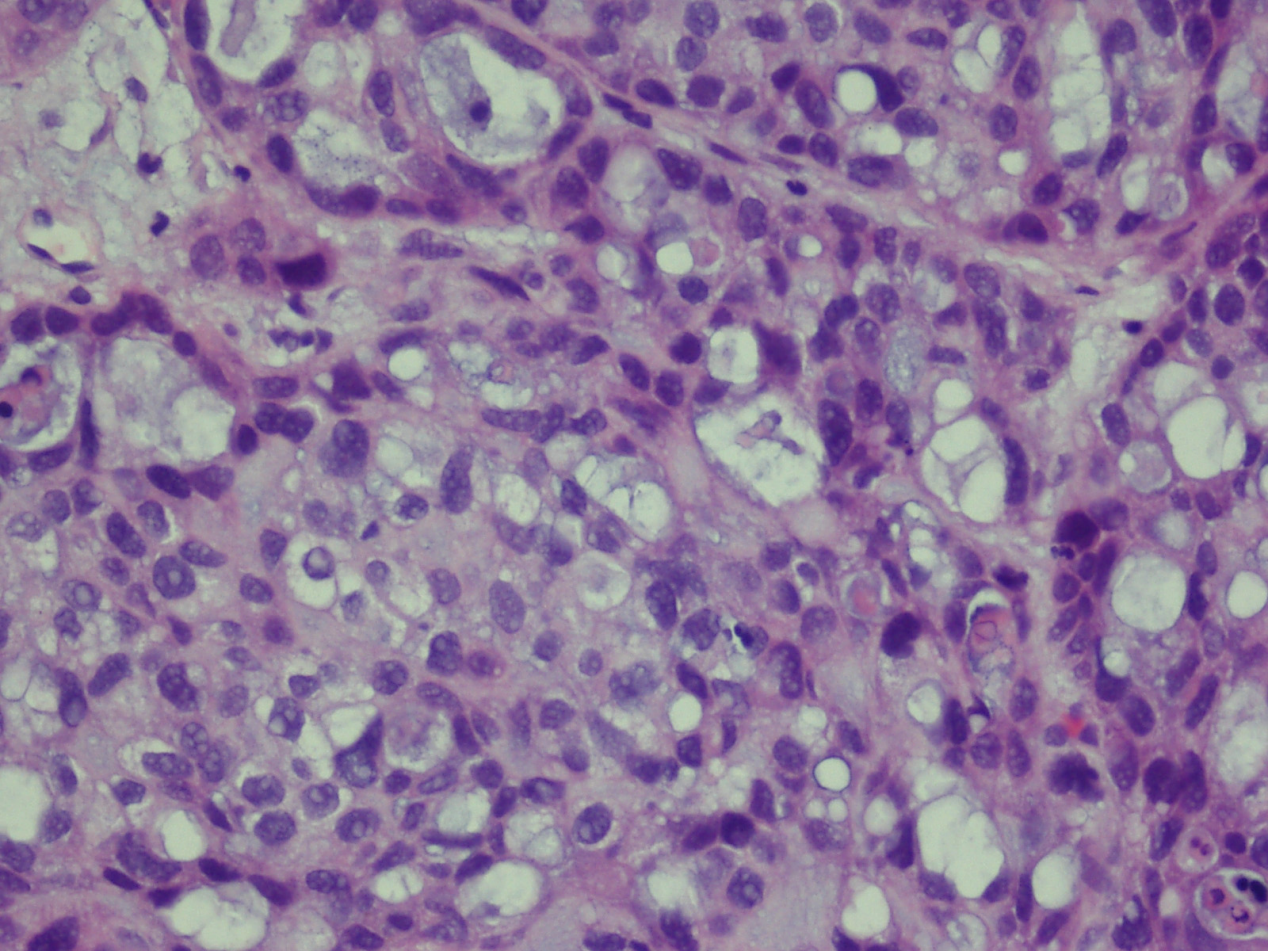
**Tumor cells were in cuboidal, columnar or hobnail shape, with abundant clear cytoplasm**.

On gross examination, the TUR tissue was fragmented and grayish white, and its texture was soft. Microscopically, most of the tumor cells were arranged in tubular glands, microcysts or fine papillae, partly in diffuse sheets or solid nests (Figure 2). The tubular glands and microcysts had different sizes and irregular shapes, which included eosinophilic and/or basophilous secretions (Figure 3). Most tumor cells were in cuboidal or columnar shape and hobnail cells were seen containing abundant clear cytoplasm (Figure 4). The pathological diagnosis was clear cell adenocarcinoma of the bladder. The muscular layer of the bladder was invaded by tumor cells. The immunohistochemical staining reveals CK7(+), CK20(+), CA125(+) and EMA(+), while PR(-), ER(-), CD10(-) and HMB(-). (Anti-CK7, Abcam, mouse monoclonal; anti-CK20, Santa Cruz, mouse monoclonal; anti-CA125, Abbiotec, rabbit polyclonal; anti-EMA, Pierce, mouse monoclonal; anti-PR, Santa Cruz, rabbit polyclonal; anti-ER, Santa Cruz, mouse monoclonal; anti-CD10, Abcam, mouse monoclonal; anti-HMB, Dako, mouse monoclonal)

**Figure 5:**
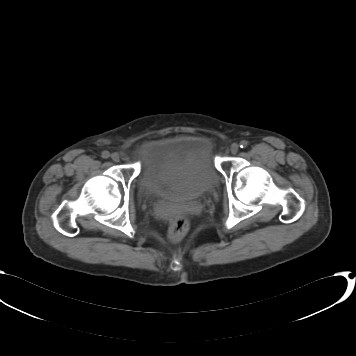
**Three months after TUR, CT showed the tumor recurrence at the posterior wall and bladder neck and the border with urethral orifice was not clear**.

Based on preoperative examination and postoperative pathological diagnosis, we determined clinical TNM stage as T2aN0M0. Postoperatively, we suggest the patient should undergo radical cystectomy. But the patient refused any partial or radical cystectomy surgeries or possible urinary diversion strongly. Then we suggested the patient try chemotherapy or radiotherapy, but the patient was still afraid of side effects and insisted receiving intravesical therapy, which is a classical postoperative treatment to prevent recurrence for transitional cell carcinoma of bladder. The postoperative therapy was mitomycin, 20 mg, once per week for 2 months. At first the patient's urinary symptoms have been relieved postoperatively. However, three months after the surgery, a recurrent tumor was found by cystoscopy. A sessile mass was found at the internal urethral orifice, located at 1-3 o'clock and 7-11 o'clock of the vesical neck through cystoscopy. CT showed that the inferior vesical thickening had become more severe, part of which was due to the presence of an irregular-shaped mass. It had wide base and rough surface, and the biggest plane was about 6.8 cm × 4.5 cm. The delimitation between internal urethral orifice and the mass was not clear (Figure 5). Enlarged lymph nodes were found in right iliac vessels and inguinal region. Although we notified her that TUR-BT could not cure her illness and recommended that radical cystectomy should be done, the patient insisted upon having TUR-BT. The pathological diagnosis was still clear cell carcinoma.

**Figure 6:**
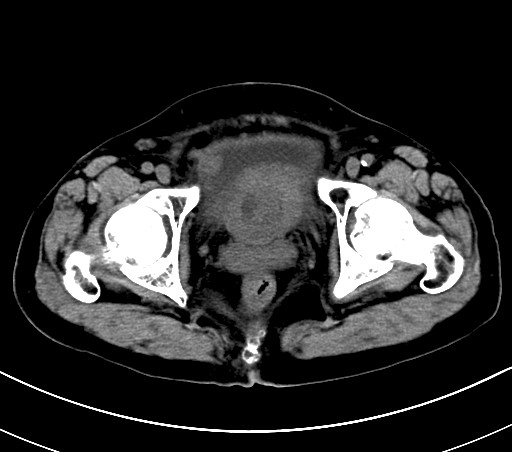
**Two months after second TUR, CT showed that the tumor recurrence again at the anterior wall and the neck, invading urethra downward**.

After the second TUR-BT, the TNM stage was evaluated as T2bN1M0. The patient still used mitomycin for intravesical therapy and rejected any cystectomy, chemotherapy and radiotherapy. Two months later, she found vulval edema and had the urinary symptoms of frequency, urgency and painful urination, complicated with dysuria. Cystoscopy and CT scan both demonstrated the carcinoma was recurrent again and that the mass had invaded the urethra (Figure 6). We considered it was too late to do radical cystectomy or any other surgeries. On the patient's strong demand, TUR-BT was done again as a cytoreductive surgery. The pathological diagnosis was the same as previously. After the third TUR-BT, the urinary symptoms such as urinary frequency, urgency, pain in urination and dysuria had little relieved. Two months later, a vaginal neoplasm and multiple cervical lymphadenectasis were found. The TNM stage has been advanced into T4aN2bM0. Following up 12 months after the last TUR-BT, she was still alive but with progression of the disease.

## Discussion

Primary clear cell carcinoma of the bladder is a very rare histological and pathological type of bladder cancer. It was first reported by Dow and Young in 1968 [5]. For a literature review we searched relevant case reports published in English language. To evaluate clinical characteristics better, the case reports that do not have any data about treatment and outcomes were not included. A total of 38 case reports (the current case and 37 others) were included in the present review (Table 1)[5-19]. Length of follow-up in these cases ranged from 8 months to 7 years.

**Table 1:** Reported cases of clear cell adenocarcinoma of the urinary bladder

**No**.	Age	Sex	Site	Treatment	Outcome	Reference
1	43	M	Right lateral wall,neck	RT,TC	DWD(1y)	[5]
2	64	F	Neck	TUR,RT	NA	[6]
3	54	F	Neck	TC	NED(2y)	[7]
4	71	F	Neck,urethra	RT	NED(2y)	[7]
5	53	F	Right ureteral orifice	TC	DWD(9m)	[7]
6	68	F	Posterior,left lateral wall	PC	NED(3y)	[6]
7	NA	NA	NA	TC	DWD(1y)	[6]
8	70	F	Neck	RS	NED(10m)	[7]
9	63	M	Left lateral wall,trigone	TC	NED(7y)	[6]
10	57	F	Neck	RT,TC	NED(18m)	[6]
11	78	F	Trigone	TUR	NED(4y)	[8]
12	61	F	Posterior wall	PC	NED(5y)	[6]
13	62	F	Left ureteral orifice	TUR	NA	[6]
14	62	F	Posterior wall,trigone	TC	NED(2y)	[6]
15	73	F	Posterior,anterior wall,neck,urethra	RS	NA	[6]
16	53	M	Posterior,anterior wall	TUR,RT	NED	[6]
17	78	F	Right lateral wall,neck	TUR,CT,TC	NA	[6]
18	72	M	Trigone,posterior,left lateral wall	TC,RT	DWD(2y)	[6]
19	35	F	Posterior wall	TC	NA	[9]
20	67	M	Trigone,posterior wall	TC	NED(14m)	[6]
21	63	M	NA	CT,RT	DWD(18m)	[7]
22	78	F	NA	RS	NED(12m)	[7]
23	50	M	NA	TUR,RS	A,P/P(63m)	[7]
24	43	M	NA	TUR	NED(30m)	[7]
25	80	M	Neck,left ureteral orifice	TUR,CT	NED(25m)	[6]
26	65	M	Anterior wall	TC,CT	DWD(18m)	[10]
27	68	F	Trigone	RS	NA	[11]
28	59	F	Trigone,neck	TC,CT	DWD(2y)	[12]
29	59	M	Posterior wall	RT	DWD(8m)	[6]
30	69	M	Diverticulum,right ureteral orifice	Diverticulectomy	NED(1y)	[13]
31	54	M	Done of bladder	PC	DWD(6m)	[14]
32	55	M	Left lateral wall	TUR,CT	DWD(18m)	[15]
33	52	F	Neck	TC,RT,CT	NED(28m)	[16]
34	68	M	NA	Tumor resection	NA	[17]
35	62	F	Neck,trigone,urethra	TUR,RS	NED(9m)	[18]
36	56	F	NA	RS	NED(10m)	[19]
37	69	M	NA	TUR	NA	[1]
38	68	F	Posterior wall,neck,urethra	TUR	A,P/P()	Current case

Abbreviations: A,P/P = alive with persistence or progression of disease; CT = chemotherapy; DWD = died with disease; NA = not available; NED = no evidence of disease; PC = partial cystectomy; RS = radical surgery; RT = radiotherapy; TC = total cystectomy.

The reported mean age at presentation was 62.2 and the median age was 63 (range, 35-80). There were more female cases than male (21 female vs 16 male), which was different from TCC. The usual presenting symptoms included gross hematuria, dysuria, recurrent UTI and suprapubic pain. Patients rarely complained of local tumor effects or pain from local spread of the tumor [6]. Based on available data, the most common locations which tumors originated from were bladder neck and posterior wall, with incidence 31.6% (12/38) and 26.3% (10/38) respectively. Other common locations included trigone, lateral wall and urethra. Unlike urothelial carcinoma, most clear cell adenocarcinomas of bladder were large, solitary masses forming papillary or sessile structures [15].

The histogenesis of CCA of the bladder remains controversial. In the older literature, tumors in most cases were designated "mesonephric adenocarcinoma", but lacking convincing evidence for a mesonephric origin [20]. Some authors believe that it arises from mullerian elements in the bladder and are histogenetically identical to the female genital tract, because in some cases the neoplasms have been associated with vesical endometriosis or have arisen in mullerian duct cysts or remnants in the bladder [9,21]. This has also be considered an explanation for the observation that female incidence is dominant. However, a recent study presented evidence for urothelial origin in most clear cell adenocarcinomas of urinary tract, despite the morphologic resemblance to mullerian-derived tumors of the female genital tract [22]. Moreover, some studies suggested that CCA may be associated with nephrogenic adenoma because they share some similar histological features [23,24].

The cystoscopy was necessary for all patients who were suspected of bladder cancer, including CCA. Because of no specific characteristics for symptoms, signs and accessory examinations compared with common urothelial carcinoma, clear cell adenocarcinoma was diagnosed mainly on histopathology. Microscopically, CCA is characterized by admixtures of tubular glands, microcysts, papillae and diffuse masses. The cells range from flat to hobnail and cuboidal with abundant, clear and glycogen-rich cytoplasm, often with significant nuclear atypia and mitotic activity [7,20]. Immunohistochemically clear cell adenocarcinoma of the bladder is strongly positive for CK7 and variably positive for CK20, which is similar to typical urothelial carcinoma. CA-125 is typically positive, which is generally accepted as a marker of mullerian differentiation [20,22].

In the literature review, initial treatment for primary clear cell carcinoma of bladder was mainly surgical resection. All but 5 patients were initially treated with a surgical resection (86.8%, 33/38). The surgical resection therapy included transurethral resection (12 patients, 31.6%), total cystectomy (11 patients, 28.9%), radical surgery (5 patients, 13.1%), partial cystectomy (3 patients, 7.9%) and other surgical resection (2 patients, 5.3%; one underwent diverticulectomy, one underwent unspecific tumor resection).

Of the 12 patients who underwent TUR-BT for their initial surgical treatment, 5 patients did not receive other therapies. One of the 5 patients presented no evidence of disease during 4-year follow-up period. Other patients underwent additional radical surgery, total cystectomy, chemotherapy and radiotherapy.

Of the 11 patients who underwent total cystectomy for their initial surgical treatment, 7 patients did not receive other therapies. The longest follow-up period with no evidence of disease was 7 years. Other 4 patients underwent adjuvant chemotherapy and/or radiotherapy, but which seems no additional effects with 2 of them died with disease in 2 years and 1 in 18 months.

Four patients underwent radiotherapy as the initial treatment and two of them received total cystectomy later. No effect of radiotherapy was observed because two cases died with disease in 8 months and 1 year, the other two alive with no evidence of disease in 18 months and 2 years [5-7]. Some patients underwent chemotherapy (including cisplatin, doxorubicin or cyclophosphamide) and/or radiotherapy (40-60 Gy total) as the adjunvant treatment, but with uncertain effects [5,6,15].

Adding 2 patients with initial radiotherapy, there were totally 35 patients underwent surgical resection. At last follow-up, a total of 17 (48.6%) patients were alive with no evidence of disease, 8 (22.9%) patients were dead with disease, 8 (22.9%) patients were no data available and 2 (5.7%) patients were alive with disease. On the basis of this review, we suggest that the recommended initial treatment for clear cell adenocarcinoma of the urinary bladder should be surgical removal.

The prognosis of clear cell adenocarcinoma of the bladder remains unclear. In general, it is more malignant than common urothelial carcinoma, but more cases and longer follow-up periods are required to elucidate these points. Lymph nodes and bone seem to be the most common metastatic sites for this disease [6,15]. In the current case, the patient was found to have multiple cervical lymphadenectasis and suspected lymphadenectasis around right iliac vessels and inguinal regions after the initial treatment. To our knowledge, the present case is the first case with CCA that underwent repeat intravesical therapy after TUR-BT and experienced tumor recurrences many times. Although there is insufficient evidence to show better prognosis of total cystectomy or radical surgery than TUR-BT, if the tumor was recurrent after TUR-BT, like the case in this paper, we believed the patient should undergo total cystectomy or radical surgery. Due to different oncologic characteristics and the prognosis of the current case, we do not think it is efficient to adopt intravesical therapy to treat CCA of bladder, which is a classical post-TUR treatment for urothelial carcinoma.

## Conclusions

In conclusion, because there are no characteristic symptoms for the clear cell adenocarcinoma of bladder, the diagnosis is mainly based on histopathology. However, once the diagnosis is determined, the radical surgery should be recommended. The radiotherapy or chemotherapy may be helpful. The post-TUR intravesical therapy is of no help for preventing recurrence, although the TUR-BT has resected all visible tumors and even reached to the muscular layer of the bladder.

## Consent

Written informed consent was obtained from the patient for publication of this Case report and any accompanying images. A copy of the written consent is available for review by the Editor-in-Chief of this journal.

## List of abbreviations

CCA: Clear Cell Adenocarcinoma; TCC: transitional cell carcinoma; TUR: transurethral resection; TUR-BT: transurethral resection of bladder tumor; UTI: urinary tract infection.

## Competing interests

The authors declare that they have no competing interests.

## Authors' contributions

JL wrote the initial draft. ZH and FJ participated in the pathology. YW, YH and CW are surgeons of the patient and help to collect clinical materials. QC conceived of the study design and helped to draft the manuscript. All authors read and approved the final manuscript.
